# Clinical investigator perspectives on patient outcomes in children with neuronopathic mucopolysaccharidosis II during intrathecal idursulfase-IT treatment

**DOI:** 10.1186/s13023-024-03147-4

**Published:** 2024-04-12

**Authors:** Karen S. Yee, David Alexanderian, Susan Martin, Bimpe Olayinka-Amao, David A. H. Whiteman

**Affiliations:** 1 Takeda Development Center Americas, Inc., Cambridge, MA USA; 2 Present affiliation: Alexion Pharmaceuticals, Inc., AstraZeneca Rare Disease, Boston, MA USA; 3 Takeda Development Center Americas, Inc., Lexington, MA USA; 4 Present affiliation: Merck, Boston, MA USA; 5 RTI Health Solutions, Ann Arbor, MI USA; 6 RTI Health Solutions, Research Triangle Park, NC USA

**Keywords:** Cognitive impairment, Mucopolysaccharidosis II, Hunter syndrome, Idursulfase, Intrathecal, Pediatric, Neuronopathic, Intellectual disability

## Abstract

**Background:**

Mucopolysaccharidosis II (MPS II) is a rare lysosomal storage disease characterized by iduronate-2-sulfatase gene (*IDS*) deficiency and downstream glycosaminoglycan accumulation. Two-thirds of patients present with neuronopathic disease and evaluating cognitive function in these patients is challenging owing to limitations of currently available tests. During the clinical development of intrathecal idursulfase (idursulfase-IT), regulatory authorities requested qualitative data to further understand the neurocognitive changes observed by the investigators through the clinical trials.

**Results:**

This qualitative study consisted of semi-structured interviews with all nine of the principal investigators who participated in the idursulfase-IT phase 2/3 (NCT02055118) and extension (NCT02412787) trials. These investigators enrolled the 56 patients with neuronopathic MPS II who qualified for the extension phase of the trial. The investigators were asked to rate the disease status of their patients. Of the 56 patients, 49 (88%) were rated as having disease that was improved/improving, stabilized or slowing progression compared with the expected outcomes with no treatment. Three patients were rated as worsening, while the remaining four patients were considered to have slowing progression or worsening disease. Similar results were demonstrated for patients aged from 3 to under 6 years at baseline, with 33 of 39 patients (85%) rated as having disease that was improved/improving, stabilized or slowing progression. Of the seven patients rated with slowing progression/worsening or worsening disease, five of them had an *IDS* variant other than missense, while two had a missense class variant. All the assigned improved/improving ratings were in patients receiving idursulfase-IT from the start of the phase 2/3 trial. Moreover, patients under 3 years of age at baseline were all rated as either improved/improving or stabilized disease. In a blinded review of patient profiles, investigators were requested to assign a disease status rating to 18 patients with large *IDS* deletions; 67% of these patients were rated as improved/improving or stabilized disease.

**Conclusions:**

This qualitative analysis provides a snapshot of clinicians’ considerations when evaluating treatment in patients with neuronopathic MPS II, compared with the expected decline in cognitive function in the absence of treatment. The results highlight the importance of robust assessment tools in treatment evaluation.

**Supplementary Information:**

The online version contains supplementary material available at 10.1186/s13023-024-03147-4.

## Background

Mucopolysaccharidosis II (MPS II; Hunter syndrome, OMIM 309900) is a rare, X-linked recessive, lysosomal storage disease characterized by deficient activity of the iduronate-2-sulfatase gene (*IDS*) and downstream glycosaminoglycan (GAG) accumulation [1, 2]. As a result, patients with MPS II experience chronic multisystemic dysfunction with a variable rate of disease progression [2].

There are many MPS II-linked *IDS* pathogenic variants [2, 3], and early evidence suggests an association between different *IDS* variant types (deletions, rearrangements, small insertions, nonsense and missense) and rates of cognitive decline [4]. Thus, knowledge of *IDS* variants may affect clinicians’ expectations of patients’ prognosis and the effect of treatment. Approximately two-thirds of patients present with neuronopathic disease; cognitive function in these patients typically slows between 2 and 4 years of age, and then declines rapidly thereafter, although there is significant variability [3–6].

Evaluating cognitive function in patients with neuronopathic MPS II is challenging owing to the limitations of currently available neurocognitive and behavioral tests [7]. To address these challenges, consensus conferences have recently been convened to align on the most appropriate neurocognitive endpoints to assess the impact of therapy in neuronopathic MPS [8, 9]. The fact that the recommendations from the same group were updated within a 3-year period highlights that this remains an evolving area of interest.

Intravenous enzyme replacement therapy (ERT) with recombinant human iduronate-2-sulfatase (idursulfase; Takeda Pharmaceuticals USA, Inc., Lexington, MA, USA) is considered to be the current standard of care for MPS II [10, 11]. Intravenous idursulfase does not cross the blood–brain barrier in sufficient therapeutic quantities to directly ameliorate neurological symptoms; nevertheless, patients with neuronopathic MPS II still benefit from the positive effect that intravenous idursulfase has on somatic outcomes [10, 12]. Various approaches for delivering ERT to the central nervous system have been evaluated, including direct delivery of idursulfase into cerebrospinal fluid (CSF) via an intrathecal drug delivery device (idursulfase-IT). The effect of idursulfase-IT on cognitive function was evaluated in children with MPS II over 52 weeks in a phase 2/3 study (HGT-HIT-094; NCT02055118) [13] and through an ongoing open-label extension trial (SHP609-302; NCT02412787) [14]. The pivotal phase 2/3 study did not meet its primary endpoint (change from baseline in Differential Ability Scales, Second Edition [DAS-II] General Conceptual Ability [GCA] score at week 52) and the study data outputs were found to be insufficient to meet the evidentiary standard to support regulatory filings.

During the clinical development of idursulfase-IT, regulatory authorities requested additional information to further understand the changes observed by the investigators in patients with MPS II over the course of the trials. We summarize the findings, based on these interviews, and anticipate that this information will be useful for the conduct of future clinical trials investigating the efficacy of other therapeutic approaches for the treatment of neuronopathic MPS II.

## Methods

### Study overview

**Fig. 1:**
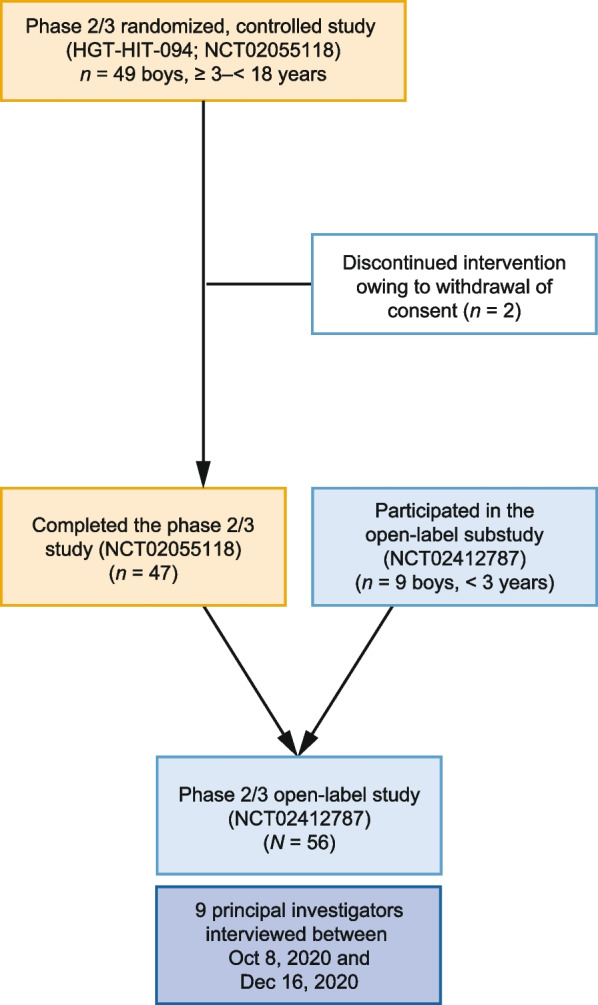
Overview of patient flow for the study and timing of interviews

This qualitative study consisted of semi-structured interviews with all nine of the principal investigators who participated in the idursulfase-IT phase 2/3 trial (HGT-HIT-094; NCT02055118) and extension trial (Fig. 1, SHP609-302; NCT02412787) [13, 14]. These investigators enrolled the 56 patients with neuronopathic MPS II who qualified for the extension phase of the trial.

Interviews were conducted at or 36 months after patient enrollment into the initial phase of the trial. The primary objective of the interviews was to understand better, from an expert perspective, the overall treatment effects observed by the clinicians that may not have been captured by the instruments (DAS-II and Vineland Adaptive Behaviors Scale [VABS-II]) used in the idursulfase-IT trials.

### Phase 2/3 idursulfase-IT trial design

The design of the phase 2/3 idursulfase-IT trial and extension have been described previously [13]. Briefly, the phase 2/3 trial was a 52-week, controlled, randomized, two-arm, open-label, assessor-blinded, multicenter study, in which eligible patients (*n* = 49) were randomized (2:1) to receive monthly idursulfase-IT 10 mg or no IT treatment, respectively, in addition to weekly intravenous idursulfase. Participants were males aged from 3 to under 18 years, with diagnosed MPS II and cognitive impairment. Idursulfase-IT was administered via a SOPH-A-PORT Mini S intrathecal drug delivery device or lumbar puncture in the event of device malfunction. In parallel to the phase 2/3 trial, a separate, 52-week, open-label, single-arm substudy was conducted in boys aged under 3 years (*n* = 9), in which all patients received monthly idursulfase-IT in addition to weekly intravenous idursulfase [15].

Patients who completed the phase 2/3 trial (*n* = 47) or substudy (*n* = 9) were eligible for enrollment into the open-label, non-randomized extension trial. All 56 patients in the extension phase received monthly idursulfase-IT in addition to weekly intravenous idursulfase. Treatment groups were defined according to treatment received before the extension phase: the early-IT group received idursulfase-IT in the phase 2/3 trial or substudy, whereas the delayed-IT group did not receive idursulfase-IT until entering the extension.

The primary endpoint in the pivotal phase 2/3 trial was change from baseline in DAS-II GCA at 52 weeks [13]. The DAS-II GCA score is a measure of verbal, non-verbal and spatial clusters of the DAS-II that provides an overall score of cognitive performance, in which higher values indicate better cognitive function (mean, 100; standard deviation [SD], 15). The DAS-II GCA score was assessed with one of two overlapping, age-based batteries in the DAS-II: the early-years battery (for children aged from 2 years 6 months to 6 years 11 months) and the school-age battery (for children aged from 7 years 0 months to 17 years 11 months). These batteries are fully co-normed for ages 5 years 0 months through to 8 years 11 months [16]. In the substudy, the Bayley Scales of Infant Development, Third Edition (BSID-III) was utilized as a measure of cognitive function until the patients reached 42 months of age, when their cognitive function was assessed with the DAS-II instrument if a neuropsychologist determined the child could be evaluated using this instrument [15]. The substudy patients were assessed with two of the five scales in the BSID-III instrument (cognitive and language). A score of 90–109 is considered to be average, 80–89 low average, 70–79 borderline, and 69 and below extremely low [17]. In both, adaptive behavior was assessed using VABS-II. The VABS-II Adaptive Behavior Composite score provides an overall measure of adaptive behavior ability in children and is a composite score of four domains (communication, daily living, socialization and motor skills [only included for children younger than 7 years]; mean, 100; SD, 15).

### Interview methodology

Verbal consent to participate and to be audio recorded was given by each investigator prior to the start of the interview. Each investigator was reminded that the interviews would be audio recorded and that these recordings would be transcribed for use in preparing a written summary report of the interviews. Interviews lasted approximately 90 min and were conducted in the investigator’s native language over the telephone by RTI Health Solutions (Research Triangle Park, NC, USA) or Global Perspectives (Calle General Yagüe 3, Oviedo, Spain). Interviews were conducted in English for investigators in the USA, UK, Canada and Australia; in Spanish for investigators in Spain and Mexico; and in French for investigators in France.

Each interview followed a semi-structured interview guide, which was developed by RTI Health Solutions in collaboration with the study sponsor, Takeda. This guide served as a topic guide that encouraged spontaneity of responses and fostered a relaxed tone throughout the interview, and was not intended to be followed in a verbatim question format. The interview started with general questions about the investigator’s experience with neuronopathic MPS II, followed by an examination of the investigator’s clinical impressions and opinion-based assessments of: (1) the applicability, relevance and interpretation of the DAS-II and VABS-II tests; (2) caregivers’ opinions on the treatment effects of idursulfase-IT; (3) the disease status/progression of their patients or patients from other sites; and (4) the general efficacy and safety of idursulfase-IT. Interviewers were trained on the interview process to ensure consistency of data collection across interviews.

### Investigators’ global ratings of disease status of their own patients

For the status/progression of patients’ disease, a rating was assigned to each patient by the investigator. Ratings were pre-defined as ‘stabilized’, ‘slowing of progression’ (relative to expected decline) or ‘worsening/anticipated decline’, based on the investigators’ knowledge of the natural history of MPS II and accounting for the expected progression of disease over time without treatment. During the interviews, however, it was evident that additional ratings were needed based on the investigators’ responses; these included ratings of ‘improved/improving’, ‘stabilized or slowing progression’ and ‘slowing progression or worsening’.

### Investigators’ blinded review of profiles of patients from other study sites

Profiles were selected from 18 patients who had *IDS* deletions that resulted in large or significant protein or structural loss because it was hypothesized that these variants were more likely to result in severe neuronopathic disease phenotypes [3, 4, 18]. Patient profiles included: (1) age at time of enrollment; (2) DAS-II and VABS-II scores; (3) clinical markers (CSF GAG levels); and (4) anti-drug and neutralizing antibody levels in the serum and CSF. Clinicians rated each patient profile as stabilized, slowing progression (compared with anticipated decline), or worsening/anticipated decline after reviewing the blinded profiles of patients from other sites. Patients from their own site were not included in their blinded set of profiles.

### Data analysis

Following completion of the interviews, RTI Health Solutions identified, characterized and summarized patterns across all interview responses. Dominating trends in each interview were identified and then compared across interviews to collate overall themes and the relative importance of different findings. Descriptive analyses are presented for the overall population and for participants under 6 years of age stratified by *IDS* genotype (missense versus other variant types), to align with post hoc subgroup analyses conducted on data from the main studies [13]. No formal statistical analyses were performed.

## Results

### Patient characteristics

Key baseline characteristics are shown in Table 1. Of the 56 patients that entered the extension study, 39 patients (70%) were aged from 3 to under 6 years of age at phase 2/3 study baseline and nine patients (16%) were under 3 years old at baseline (substudy group). Twenty-six patients (46%) had a missense class variant, while the remaining 30 patients had other *IDS* variant types. Detailed patient baseline characteristics have been previously reported [13].

**Table 1:** Patient baseline characteristics

	**Patients (*n* = 56)**
Median (range) age at study baseline, years	
Phase 2/3 study	4.6 (3.1, 14.3)
Substudy	2.6 (1.4, 3.0)
Age category at study baseline, n (%)	
≥ 6 years	8 (14%)
3– < 6 years	39 (70%)
< 3 years	9 (16%)
Age at time of interview with investigator, years	
Median (range)	9 (5–18)
*IDS* genotype category, n (%)	
Missense class variant	26 (46%)
Not missense class variant	30 (54%)
Treatment group,^a^ n (%)	
Early IT	41 (73%)
Delayed IT	15 (27%)

*IDS* iduronate-2-sulfatase gene, IT intrathecal

^a^The early-IT group comprised patients who received idursulfase-IT in the phase 2/3 trial and extension, and patients who received idursulfase-IT during the substudy and extension. The delayed IT group comprised patients who initiated idursulfase-IT in the extension and did not receive idursulfase-IT during the phase 2/3 trial

### Investigators’ global ratings of disease status of their own patients

**Fig. 2:**
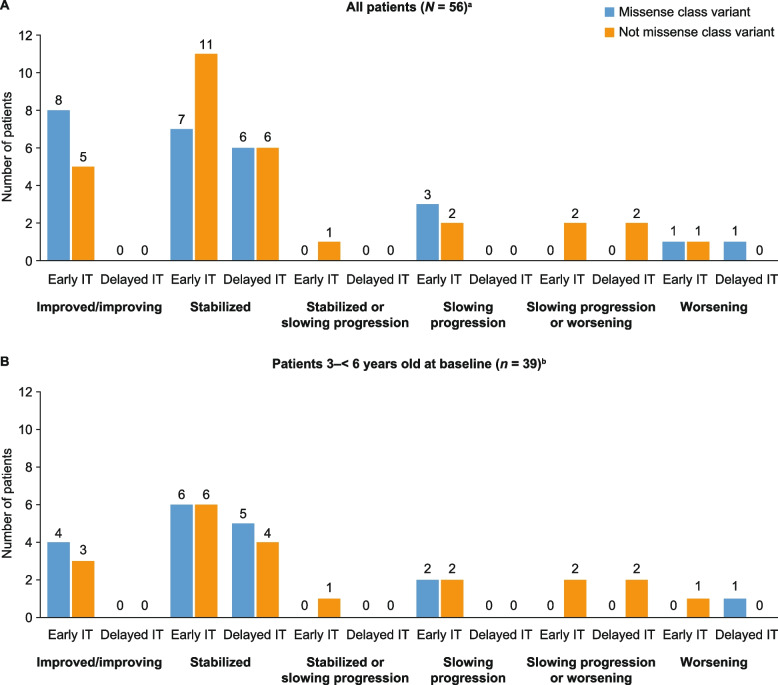
Investigators’ global disease status/progression ratings for patients at their own study site. Patients in the early-IT group received idursulfase-IT in the phase 2/3 and extension studies; patients in the delayed-IT group initiated treatment with idursulfase-IT in the extension study. ^a^Includes nine patients aged under 3 years enrolled in the substudy as part of the early-IT population. ^b^Excludes nine patients aged under 3 years enrolled in the substudy. IT, intrathecal

Overall, 49 of the 56 patients were categorized by the investigators as having disease that was improved/improving, stabilized, or stabilized or slowing progression (Fig. 2A). Notably, all 13 children showing improvement were in the early-IT treatment group and all were under 6 years of age at baseline. The proportion of patients who had not worsened was higher in the early-IT group (37/41; 90%) than in the delayed-IT group (12/15; 80%). A similar pattern was seen for the subgroup of 39 patients aged from 3 to under 6 years at baseline in the phase 2/3 pivotal trial (Fig. 2B). In these patients, the proportion who had not worsened was higher among those with missense *IDS* variants (17/18; 94%) than for those with *IDS* variants other than missense (16/21; 76%).

Four patients were rated as having slowing progression or worsening disease (all were variants other than missense; early-IT group, *n* = 2, delayed-IT group, *n* = 2), and three patients were categorized as having worsening disease (two missense class variants and one other variant; early-IT group, *n* = 2, delayed-IT group, *n* = 1) (Fig. 2A). The three patients with worsening disease included one patient aged under 6 years at baseline who did not receive idursulfase-IT until participation in the extension study (delayed-IT group); one patient over 8 years of age treated with idursulfase-IT in the phase 2/3 trial and extension study (early-IT group); and one patient under 6 years of age in the early-IT group. Of the nine patients under 3 years of age and included in the substudy, six were rated as having improved/improving disease (missense class variant, *n* = 4; variant other than missense, *n* = 2), while the remaining three (all with variants other than missense) were rated as stabilized.

**Fig. 3:**
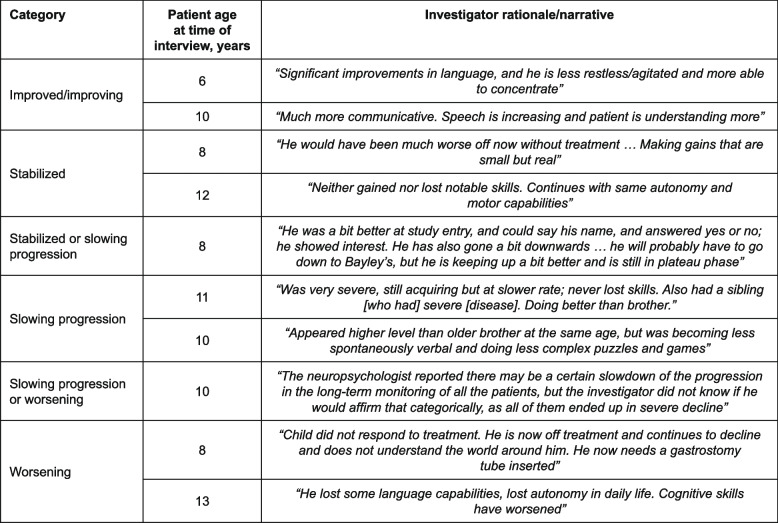
Narratives from investigators describing patients for each disease status/progression ratings

All clinical investigators (*N* = 9) from the idursulfase-IT phase 2/3 trial participated in the interviews. In their description of their patients’ disease progression, three of the investigators drew upon their experiences in treating the sibling of the patient; for example, comparing language skills or toilet training between the treated and untreated brothers at similar ages. Narrative examples from the investigators describing patients for each of the different ratings are shown in Fig. 3. Overall, despite the substantial variability reported in the description of disease status and progression of patients, there were some common disease outcomes that were considered by the investigators for their chosen ratings. Patients classified as having disease that was improved/improving were commonly described as interactive, verbally skilled and toilet trained. Those rated as ‘stabilized’ were characterized by a stabilized trajectory within a wide spectrum of disease severity, different from those with a stabilized or slowing progression, or slowing progression disease, who generally showed small improvements in skill acquisition but at a slower rate. Patients were classified as having slowing progression/worsening or worsening disease when cognitive skills declined, neutralizing antibodies were present or certain slowdown of the progression in the long-term monitoring was observed. A complete list describing the rationales given by the physicians for their ratings is given in Supplementary Table 1.

### Investigators’ blinded review of profiles of patients from other study sites

Of the 18 patients with large *IDS* deletions from other study sites, 17 were aged under 6 years at baseline and four participated in the substudy (under 3 years of age). Based on the blinded profiles providing information on patient age at time of enrollment, DAS-II and VABS-II scores, CSF GAG levels and serum/CSF antibody levels, three of the investigators found the available information to be insufficient to make a disease status/progression rating. Several investigators highlighted the need to see patients in person to provide an appropriate and informed assessment.

**Fig. 4:**
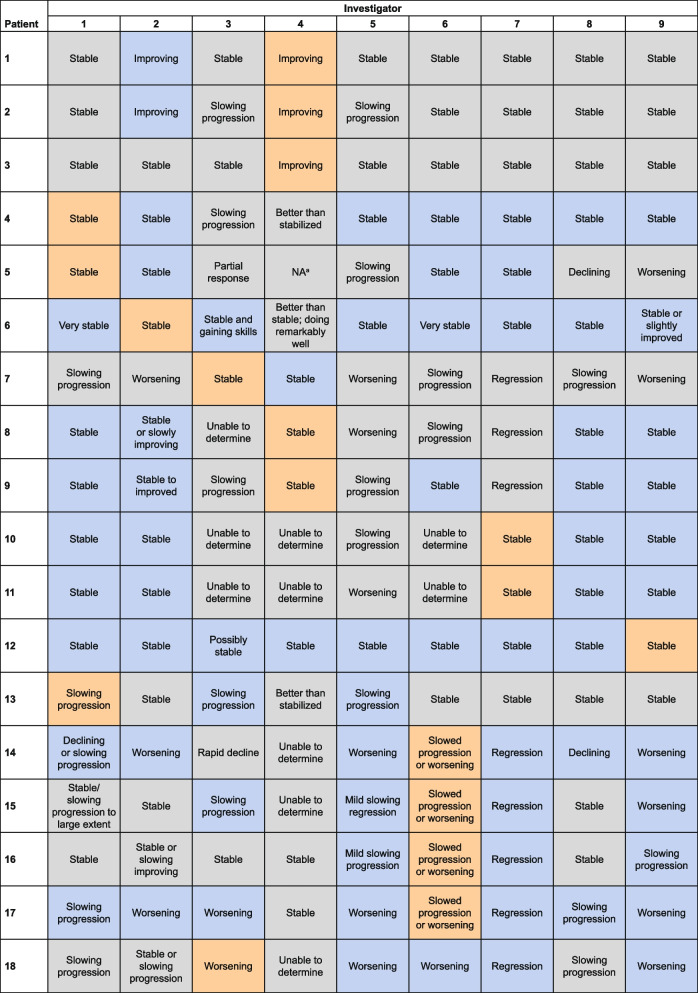
Investigators’ rating of disease status/progression based on blinded review of patients from other sites. Orange ratings are from the investigator treating the patient. Blue highlighting indicates blinded ratings the same/similar to the treating investigator. Gray highlighting represents blinded ratings different to the treating investigator. ^a^This patient transferred from Investigator 4 to Investigator 1; neither conducted a blinded review. NA, not available

There was clear variability in the ratings between investigators (Fig. 4). For one patient, whose disease was considered to be ‘stable’ by his own investigator and three of the blinded ratings, ratings ranged from partial response by one investigator to worsening/declining in two. For another patient, whose disease was considered to be ‘slowly progressing’ by his own investigator and two of the blinded ratings, five ratings categorized the disease as ‘stable’ and one as ‘better than stabilized’. There was only one patient with the same rating across all investigators (rated ‘stable’ by eight and ‘possibly stable’ by one). There were 12/18 patients (67%) in whom the investigator’s own rating was improving or stable and for 10/12 of these patients (83%) at least four of the eight other investigators also rated the disease as improved or stabilized. There were six patients in whom the investigator’s own rating was slowing/slowed progression or worsening and for 4/6 patients (67%), at least four of the eight other investigators provided a similar rating.

### Investigators’ views on the clinical utility of DAS-II and VABS-II scores

During the idursulfase-IT trial, all investigators reported that they collaborated with neuropsychologists, or other appropriately trained individuals, who administered and interpreted the DAS-II and VABS-II assessments. The investigators noted that, outside of this trial, they would not use the DAS-II and VABS-II tools, and therefore do not consider themselves to be experts in interpreting the scores from these instruments. However, based on their personal views, the investigators reported certain limitations of these tools.

Reported challenges associated with using the DAS-II in children with MPS II included the effect of physical limitations (such as carpal tunnel syndrome affecting drawing skills) on test outcomes as well as lack of cooperation from the child, with low attention spans and frustration thresholds. Investigators reported that DAS-II scores do not fully reflect the improvements that might be seen in clinical practice, and therefore treatment benefits to both patients and caregivers may be missed. They also noted their interpretations of DAS-II results were limited by the inability to compare scores against an untreated patient. One investigator noted that the test does not translate culturally, particularly from a language perspective.

Only one investigator reported administering the VABS-II themselves and several did not have any feedback on the instrument. The importance of VABS-II as a parent-reported tool was noted, although the main concern highlighted by investigators was that caregivers may be overly optimistic when answering questions, leading to higher ratings than are appropriate. One investigator described the discordance between the VABS-II scores and the clinical opinion of the treating investigator regarding the patient’s skills as ‘surprising’. Two investigators acknowledged that, unlike some tests that are conducted during a single visit, the VABS-II provides more of a longitudinal perspective, unaffected by whether the patient was having a ‘good day’ or ‘bad day’ at the time of the assessment. The majority of investigators noted that both DAS-II and VABS-II scores were unlikely to be affected by recall bias when the assessment was repeated.

## Discussion

As per the request of regulatory authorities to gain a better understanding of clinician assessments of cognitive abilities in MPS II patients, this qualitative analysis aims to provide context to the neurocognitive test results from the idursulfase-IT phase 2/3 trial and extension in patients with neuronopathic MPS II, as experienced first-hand by the nine principal investigators who enrolled patients in the trials. Accordingly, the investigators were asked to rate the disease status of their patients. Of the 56 patients assessed by the investigators, 49 (88%) were rated as having disease that was improved/improving, stabilized or slowing progression compared with the expected outcomes with no treatment. Only three patients were rated as worsening, while the remaining four patients were considered to have slowing progression or worsening disease. Similar results were demonstrated for patients aged from 3 to under 6 years at baseline, with 33 of 39 patients (85%) rated as having disease that was improved/improving, stabilized or slowing progression. All the assigned improved/improving ratings were in patients in the early-IT group. Moreover, patients under 3 years of age at baseline from the substudy were all rated as either improved/improving or stabilized disease. After many years of extensive review and regulatory discussions, the idursulfase-IT data were found to be insufficient to meet the evidentiary standard to support regulatory filings. However, overall, the general impression of the clinical investigators was that idursulfase-IT treatment was beneficial in some patients, having the potential to alter the course of MPS II disease, and with patients treated earlier (at a younger age or earlier in the disease process) experiencing the greatest benefit. Thus, idursulfase-IT will continue to be made available to patients who are currently enrolled in the ongoing open-label extension studies until an alternative approved treatment option is available to address cognitive symptoms.

There is limited evidence exploring the predictive effect of *IDS* genotype on phenotype severity and treatment response in patients with MPS II [4]. Notably, of the seven patients rated with slowing progression/worsening or worsening disease, five of them had a variant other than missense. This might be expected given that patients with variants other than missense, particularly deletions, would be expected to exhibit more rapid cognitive decline [4], and therefore a more difficult-to-treat phenotype, compared with patients with missense class variants. Indeed, multiple studies have shown that *IDS* variants that disrupt idursulfase expression – such as large deletions, frameshifts and recombinations – are associated with neuronopathic phenotypes in MPS II, whereas variants impairing activity but still allowing the enzyme to be expressed may result in non-neuronopathic clinical presentations [4, 19, 20], in line with our findings.

In a blinded review of patient profiles, investigators were requested to assign a disease status rating to 18 patients with large *IDS* deletions; 67% of these patients were rated with improved/improving or stabilized disease. The results were varied with discordance among ratings of nearly all patients – only one patient had the same rating across all investigators. The blinded review was based on several parameters including age at time of enrollment, DAS-II and VABS-II scores, and anti-drug/neutralizing antibody levels and, while these outcomes helped to inform the rating, investigators stressed the need to see a patient in person to give a fully informed prognosis.

To understand the challenges that clinicians may experience in interpreting the results and clinical implications of neurocognitive tests, the investigators were also asked to provide feedback on the use of the two assessment tools used in the pivotal idursulfase-IT trial, the DAS-II and VABS-II. The investigators highlighted the challenge of testing children with neuronopathic MPS II who often have complex physical and behavioral problems, and the challenges in capturing improvements that are still below the expected rates of healthy children. Indeed, some investigators commented that the benefit obtained with idursulfase-IT in patients with MPS II may not be measured adequately with the assessment tools, and that the clinical impression that can be obtained by knowing the patient is more important than the test scores themselves. It was also observed that, in order to interpret DAS-II data, developmental data from untreated children of an equivalent age would be helpful to understand the natural history of MPS II scores over time, and to plot the cognitive trajectory of the disease. As such, norm-based scores may be limited in their ability to capture whether the child is still developing, especially in the younger age groups [7, 21]. Based on investigator observations, and as supported by other studies [21–23], the selection of appropriate cognitive and behavioral assessment tools for future studies is of critical importance. The interviewed investigators stated that they do not use either the DAS-II or VABS-II assessment tools in clinical practice and generally did not believe the tests alone were an accurate reflection of their clinical opinion.

Overall, investigators provided positive, opinion-based evaluations of idursulfase-IT treatment in MPS II compared with outcomes expected without treatment based on the information available to them. Most patients had disease that was considered by the investigators to have improved or stabilized following idursulfase-IT treatment. The physicians considered that patients who received treatment at a younger age and earlier stage in the disease process had the greatest benefit. While the primary pivotal study did not meet its primary endpoint, there was a non-significant trend towards cognitive benefit of idursulfase-IT in patients under 6 years of age [13, 14]. This is consistent with the recent findings showing that early ERT initiation is beneficial for patients with MPS II, especially those younger than 3 years of age [14, 24].

At the time of the primary study, both DAS-II and VABS-II were recommended measures of cognitive function and adaptive behavior, respectively, for multinational trials evaluating the effect of treatment in MPS II [8]. A recent 2020 amendment to these recommendations has favored the use of the Kaufman Assessment Battery for Children Nonverbal Index, Second Edition because it can be administered with minimal confounding from disease-specific issues such as hearing loss and musculoskeletal issues that inhibit fine motor skills, and because the DAS-II is only available in English and Spanish, which limits its utility in multinational trials [9]. There is a need for improved standardization of implementing tests to reduce the variability of test outcomes, ensuring adequate training of the examiner so that the abilities of the patient are considered [7]. As we await these developments, a combination of expert evaluation of validated tests, caregiver assessment, and clinician’s experience and judgement may provide an improved picture of disease progression and treatment response in patients with MPS II.

Study limitations include inherent drawbacks to qualitative analyses, such as the subjective nature of opinion-based assessments, recollection bias and the lack of formal statistical analysis, owing to the qualitative nature of the data and the small sample size. Clinical investigators and patients (who were not blinded to randomization) were participating in a clinical trial and our findings, therefore, may not fully reflect real-life clinical practice. Whilst the lack of a structured process to conduct the interviews may have resulted in the collection of subjective outcomes, the results of this work provide clinical insights on the interpretation of the overall clinical benefits of idursulfase-IT treatment, which not only include clinical outcomes but also additional considerations reported by healthcare providers, patients and caregivers. Nevertheless, future studies will benefit from providing a benchmark for the interviewers, in order to have more transferable and generalizable outcomes. After many years of extensive review and regulatory discussions, the idursulfase-IT data were found to be insufficient to meet the evidentiary standard to support regulatory filings. However, idursulfase-IT will continue to be made available to patients who are currently enrolled in the ongoing open-label extension studies until an alternative approved treatment option is available to address cognitive symptoms.

## Conclusions

In conclusion, our qualitative analysis provides a snapshot of clinicians’ considerations when evaluating treatment in patients with neuronopathic MPS II, compared with the expected decline in cognitive function in the absence of treatment. The results highlight the importance of robust assessment tools in treatment evaluation.

### Supplementary Information

**Supplementary Material 1.**

## Data Availability

De-identified individual participant data from this particular report will not be shared as there is a reasonable likelihood that individual patients could be re-identified (due to the limited number of study participants and the narratives provided).

## Abbreviations

BSID-III: Bayley Scales of Infant Development, Third Edition
CSF: Cerebrospinal fluid
DAS-II: Differential Ability Scales, Second Edition
ERT: Enzyme replacement therapy
GAG: Glycosaminoglycan
GCA: General Conceptual Ability
*IDS*: Iduronate-2-sulfatase gene
IT: Intrathecal
MPS II: Mucopolysaccharidosis II
SD: Standard deviation
VABS-II: Vineland Adaptive Behavior Scales, Second Edition
