# Supplementary material for: Clinical investigator perspectives on patient outcomes in children with neuronopathic mucopolysaccharidosis II during intrathecal idursulfase-IT treatment
Source: Orphanet J Rare Dis. 2024 Apr 12;19:158. doi: 10.1186/s13023-024-03147-4 (PMC11015594; doi:10.1186/s13023-024-03147-4)
Supplement: Supplementary file 1 — Supplementary Material 1. [file 13023_2024_3147_MOESM1_ESM.docx]

**Clinical investigator perspectives on patient outcomes in children with neuronopathic mucopolysaccharidosis II during intrathecal idursulfase-IT treatment**

**Karen S. Yee, David Alexanderian, Susan Martin, Bimpe Olayinka-Amao, David A. H. Whiteman**

**Supplementary materials**

**Table S1.** Investigators’ rationale for their global disease status/progression ratings for patients at their own study site

| **Rating** | **Age at time of interview, years** | **Rationale for response** |
| --- | --- | --- |
| **Missense genotype variants** | | |
| Improved | 7 | Significant achievements (reading, writing), normal socialization, playing football [soccer in the US], normal autonomy for his age, great progress in language. |
| Improving | 8 | This child is the most obvious good outcome. He is still learning at age 8, which is just remarkable. MD would have expected him to be declining and losing skills. |
| Improving | 11 | Younger sibling of [another patient in the studies], but this child is a star. Almost like normal child, toilet trained, plays baseball, is a pitcher, winning pitcher of OT game, age 9, printed his name on the baseball and gave it to [investigator], almost close to age for grade, some attention issues, IQ in 80’s gaining at a normal rate for age. |
| Improving | 10 | Much more communicative. Speech is increasing and patient is understanding more. Hard to tell if gaining skills at a normal rate. |
| Improving | 10 | Very shy early on and little talking. He has come a long way. Talks much more, has a lot more nonverbal skills, and is interactive and bossy. Likes to play with tools. Usually by age 10 these patients would not be interacting with others. Just looking at GCA, he may be fairly impaired. |
| Improving | 7 | Sibling to [another patient in the studies]. This is another star. Toilet trained, knows his colors and numbers, talkative. Clearly benefiting. |
| Improving | 7 | Close to being a star. Doing remarkably well. Much more mature. Toilet trained. He has 2 older brothers with MPS II and they are much more impaired. They were not like him at this age. |
| Improving | 9 | Understands more. Starting to be toilet trained. Making progress but more behavior issues. He finds it hard to wait. He has a cousin who was older and now deceased from MPS II and family reports this child is doing much better than cousin when at same age. |
| Stabilized | 9 | Really done well. Knows older brother, who is very severe and did not qualify for entry. This child doing really good. |
| Stabilized | 9 | He’s a star. Great improvement: close to being a normal child (10 year old). In a normal classroom, plays baseball. |
| Stabilized | 10 | Almost thought might be attenuated, making forward progress. He's got complex speech, toilet trained. He's very social kid, has no behavioral issues. |
| Stabilized | 9 | Back at home site, no issues. |
| Stabilized | 9 | Done well. |
| Stabilized | 10 | Was in control group, and seemed to improve skills after starting on treatment. On treatment gained skills such as toilet training, increased sentence length, more verbal and telling jokes. |
| Stabilized | 8 | Engages with staff and enjoys playing with toys. Language is still bilingual. Has good spatial awareness, remembering where things are and where to go. |
| Stabilized | 14 | Patient has slightly improved learning, although it is at a slower rate than a typical child of his age. Based on where he started and his phenotype, a slow decline was expected, if untreated, and instead [the investigator] considered this patient very stable. |
| Stabilized | 14 | Although significantly impaired at baseline and developed meningitis after first dose, has not lost any skills and maybe gained a little bit. |
| Stabilized | 9 | Nontreated group for first year, lost a lot of skills. Once treated not losing anything, more like [another patient in the studies] but a little better than [another patient in the studies]. This mom also thinks that near the end of the dosing period, not doing as well. |
| Stabilized | 8 | Not making a lot of progress, maybe just a little. Not losing skills. |
| Stabilized | 9 | The disease did not progress. The investigator asks them to draw and to write their names at each visit, and their ability to complete these tasks has been maintained. |
| Stabilized | 10 | The disease did not progress. The investigator asks them to draw and to write their names at each visit, and their ability to complete these tasks has been maintained. |
| Slowing progression | 11 | Was very severe, still acquiring but at slower rate; never lost skills. Also had a severe sibling. Doing better than brother. |
| Slowing progression | 12 | Still increasing sleep disturbance on study, lost spoken words. Was still toilet trained. Had lowest DQ of any patients at start. Exited study at approximately age 11.5, family felt the stress of the procedures and evaluations outweighed continuing therapy. |
| Worsened | 13 | He lost some language capabilities, lost autonomy in daily life. |
| Worsening | 9 | Regressing; anticipate further decline. Has neutralizing antibodies. Cognitive skills have worsened. |

| **Rating** | **Age at time of interview, years** | **Rationale for response** |
| --- | --- | --- |
| **Non-missense genotype variants** | | |
| Improved | 6 | Significant improvements in language, and he is less restless |
| Improving | 10 | Child has big deletion arrangement. He is gaining skills and loves puzzles and is good at them. Mother reports that he does not do as well at week 4 as he does in earlier weeks after dosing. Testing at week 4. Better verbal than nonverbal skills. |
| Improving | 10 | Became toilet trained 6 months ago. This is just unheard of in MPS II. Talkative and gaining skills but not at a normal rate. Most at age 10 would not be interactive if untreated. |
| Improving | 7 | Another child with a lot of medical problems. Child has made significant progress. Child challenges you while talking. Clearly benefiting. Mother reports sees a significant difference to nephews who also have MPS II. |
| Improving | 8 | Big genetic deletion; therefore, should be profoundly impaired. He is inquisitive, making progress toward toilet training. Delayed but improving and gaining skills but not at a normal rate. |
| Stabilized | 8 | He would have been much worse off now without treatment. He is developing new skills, language is coming along, behavior is improving, improvement in receptive and expressive language, and knows when to modify behavior. All of this is unheard of. He is toilet trained and attends school. |
| Stabilized | 5 | Doing awesome, really fine, has severe mutation and antibodies, clinically doing very well. |
| Stabilized | 10 | Goes to school/talks/walks. |
| Stabilized | 9 | Doing really well. Pretty |
| Stabilized | 12 | Neither gained nor lost notable skills. Continues with same autonomy and motor capabilities. |
| Stabilized | 11 | Gaining skills at a good rate. Still in a mixture of general and special education in school. Good social skills, interacting with peers and siblings. Improving life skills; could buy lunch at the cafeteria without parental assistance. Completing increasingly complex Lego structures and following instructions. |
| Stabilized | 9 | Gained skills, but possibly slower than other patients. Toilet trained, hearing and language improved. More socially appropriate behavior and cooperation with procedures. |
| Stabilized | 11 | Reading at close to grade level. Increased attention span. Toilet trained. |
| Stabilized | 13 | Anxiety improved, went from needing sedation to being able to do procedures without medication or anesthesia. Better control with voiding, able to hold longer and anticipate needing to use the bathroom. |
| Stabilized | 18 | He is very stable, able to fully communicate. MD did not see any regression over 5 years. Patient is toilet trained and in special need’s classroom. |
| Stabilized | 7 | Continues to gain developmental skills. He is toilet trained, moves around, and plays. He is delayed and in a special need’s classroom. |
| Stabilized | 7 | He has not improved but there has not been any regression either. He has severe attention deficit disorder and hyperactivity. He is nonverbal but is toilet trained. He is in a special need’s classroom. |
| Stabilized | 11 | He has not gained or lost skills. When he entered the study he was rapidly declining and MD expected him to have been in a wheel chair, have a gastrostomy tube, and be experiencing seizures by now. None of those have happened. MD finds his stabilization remarkable because we was in a rapidly declining phase. Overall function has not changed. |
| Stabilized | 9 | Older brother to [another patient in the studies]. Child has a lot of medical problems and is anxious (“medical trauma”). No language skills and hard to assess. Gaining a little skills. |
| Stabilized | 7 | Also has anxiety but not as bad as sibling [another patient in the studies]. Making more progress than his brother but not a lot. Mother reports that she likes what is happening. |
| Stabilized | 12 | The disease did not progress. The investigator asks them to draw and to write their names at each visit, and their ability to complete these tasks has been maintained. |
| Stabilized | 10 | The disease did not progress. The investigator asks them to draw and to write their names at each visit, and their ability to complete |
| Stabilized or slowing progression | 8 | He was the youngest patient at our site, at 3 years and 8 months old, when he was included in the study. He was a bit better at study entry, and could say his name, and answered yes or no; he showed interest. He has also gone a bit downwards; in fact, the neuropsychologist says that now that he is 7 years old, he will probably have to go down to Bayley’s, but he is keeping up a bit better and is still in plateau phase. |
| Slowing progression | 9 | Continues gains but at slower rate. |
| Slowing progression | 7 | More somatic issues, bad airway problems, device issues. Continuing to acquire skills. A little bit more severe than others. |
| Slowing progression or worsening | 10 | The neuropsychologist reported there may be a certain slowdown of the progression in the long-term monitoring of all the patients, but the investigator did not know if he would affirm that categorically, as all of them ended up in severe decline. |
| Slowing progression or worsening | 9 | The neuropsychologist reported there may be a certain slowdown of the progression in the long-term monitoring of all the patients, but the investigator did not know if he would affirm that categorically, as all of them ended up in severe decline. |
| Slowing progression or worsening | 10 | The neuropsychologist reported there may be a certain slowdown of the progression in the long-term monitoring of all the patients, but the investigator did not know if he would affirm that categorically, as all of them ended up in severe decline. |
| Slowing progression or worsening | 10 | The neuropsychologist reported there may be a certain slowdown of the progression in the long-term monitoring of all the patients, but the investigator did not know if he would affirm that categorically, as all of them ended up in severe decline. |
| Worsening | 8 | Child did not respond to treatment. He is now off treatment and continues to decline and does not understand the world around him. He now needs a gastrostomy tube inserted. He has all of the antibodies. Difference between him and patient above is night and day. |

DQ, developmental quotient; GCA, General Conceptual Ability; IQ, intelligence quotient; MD, medical doctor; MPS II, mucopolysaccharidosis II
